# Spontaneous closed zone IV rupture of flexor digitorum profundus tendon to the fifth finger

**DOI:** 10.1080/23320885.2018.1522960

**Published:** 2018-10-09

**Authors:** Harry Whitehouse, Jeffrey C. Y. Chan, Matthew James

**Affiliations:** Department of Plastic, Reconstructive and Hand Surgery, St. Thomas’ Hospital (Guy’s and St Thomas’ NHS Foundation Trust, London, UK

**Keywords:** Rupture, Spontaneous, Flexor Digitorum Profundus, Zone IV

## Abstract

We present a case of spontaneous closed flexor digitorum profundus (FDP) tendon rupture of the fifth finger occurring in the carpal tunnel region (Zone IV) of the hand. A review of literature illustrates that spontaneous rupture of FDP in Zone IV is extremely rare and we wish to highlight this.

## Introduction

Boyes et al. devised the term spontaneous tendon rupture to describe intra-tendinous rupture in the absence of intrinsic or extrinsic pathology i.e. without a clear underlying mechanism such as rheumatoid arthritis, bony abnormality, infection, tumour or trauma [1].

It is a rare phenomenon with the underlying pathology remains largely unknown but changes have been observed at a microscopic level [2]. It is important to have an appreciation of these rare injuries to provide the best patient care by ensuring appropriate investigations are performed and thus avoiding unnecessary incisions and scarring.

## Case report

A 46-year-old male, right hand dominant, information technology manager, was referred to clinic with a flexor tendon deficit in the right little finger.

**Figure 1.**
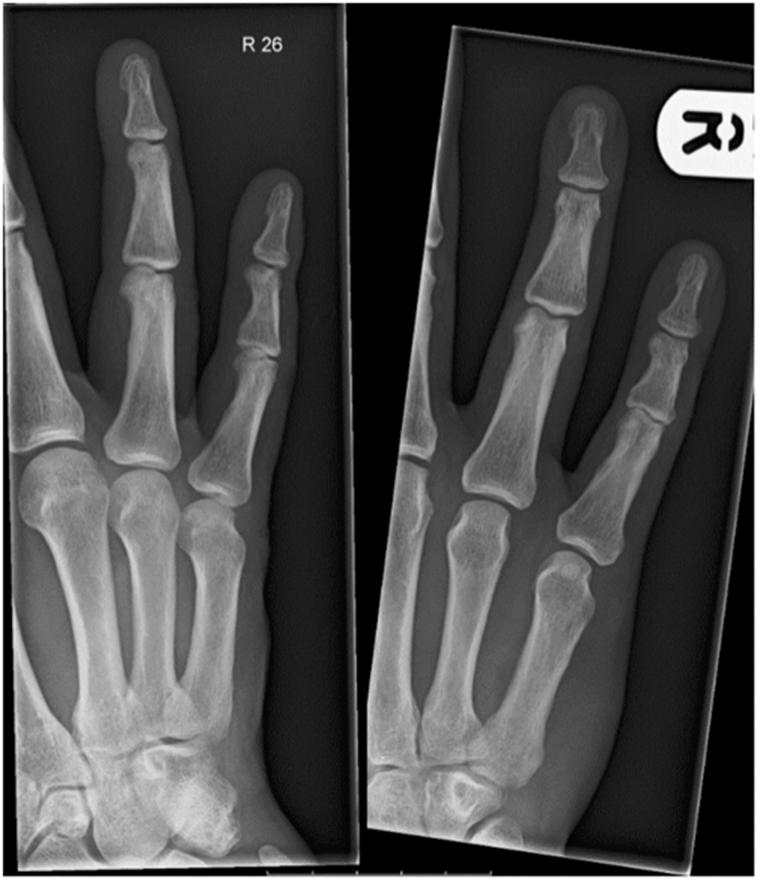
Radiograph right hand showing no bony irregularities.

He described an onset of pain and cramping within the palm, whilst pulling open a door with his right hand. The pain instantaneously resolved but he was left with an immediate inability to flex the right little finger at the distal interphalangeal joint (DIPJ). Prior to this, he had not experienced any difficulty or discomfort on flexing this finger. The patient denied any history of trauma or previous injections in the hand or wrist. He was otherwise healthy and a non-smoker. Blood tests and radiographs were unremarkable (Figure 1).

On clinical examination, there was no bruising but there was slight tenderness along the 5th finger and distal palm and he was unable to flex his DIPJ. A closed FDP tendon avulsion injury, at the bony insertion, was suspected and surgical exploration and repair was planned.

Intra-operatively, the FDP tendon was found to be intact in Zones I, II and III. Despite this, the tendon was lax with absence of tenodesis effect. The wrist was explored next on the suspicion of rupture at the musculotendinous junction.

**Figure 2.**
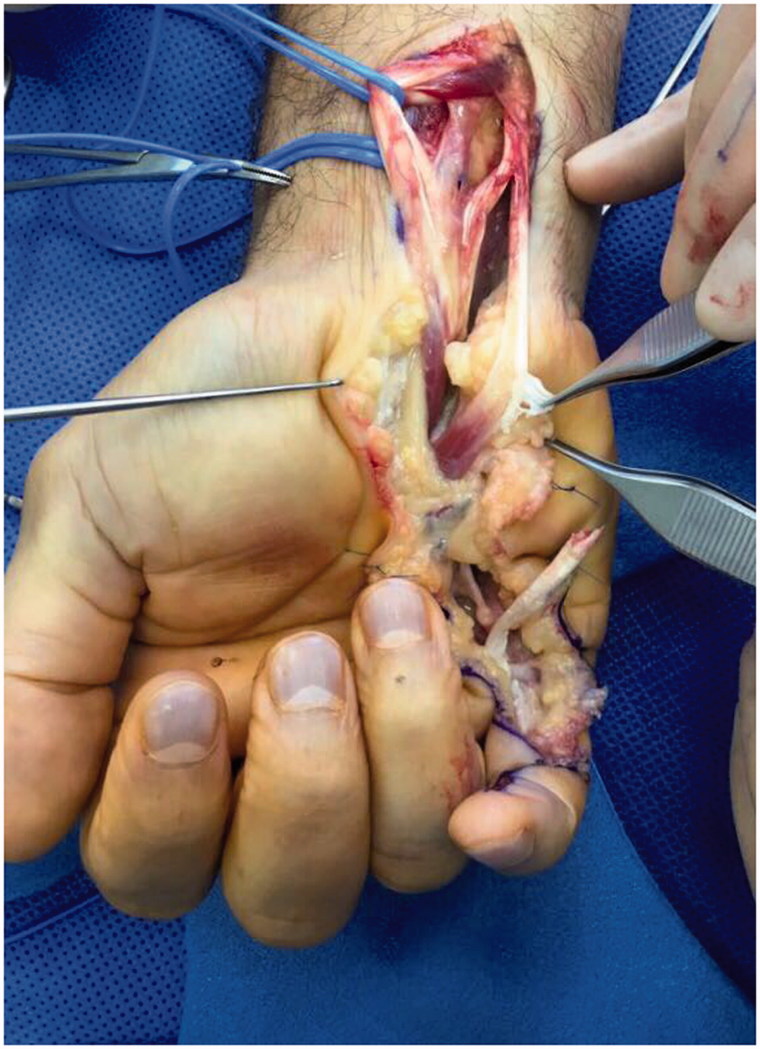
Exploration of hand showing rupture of FDP at origin of the lumbrical.

FDP was found to be intact but lax and was not activating the distal FDP tendon. The carpal tunnel was then explored, eventually identifying the FDP rupture site at the origin of the lumbrical muscle. There were no signs of synovitis or attrition and no sharp edges within, the carpal tunnel (Figure 2). Except for the rupture site, the tendon substance and lumbrical muscle were normal. The rupture was repaired with a four-strand core Adelaide repair using 3/0 Prolene and simple running epitendinous repair with 5/0 Prolene.

**Figure 3.**
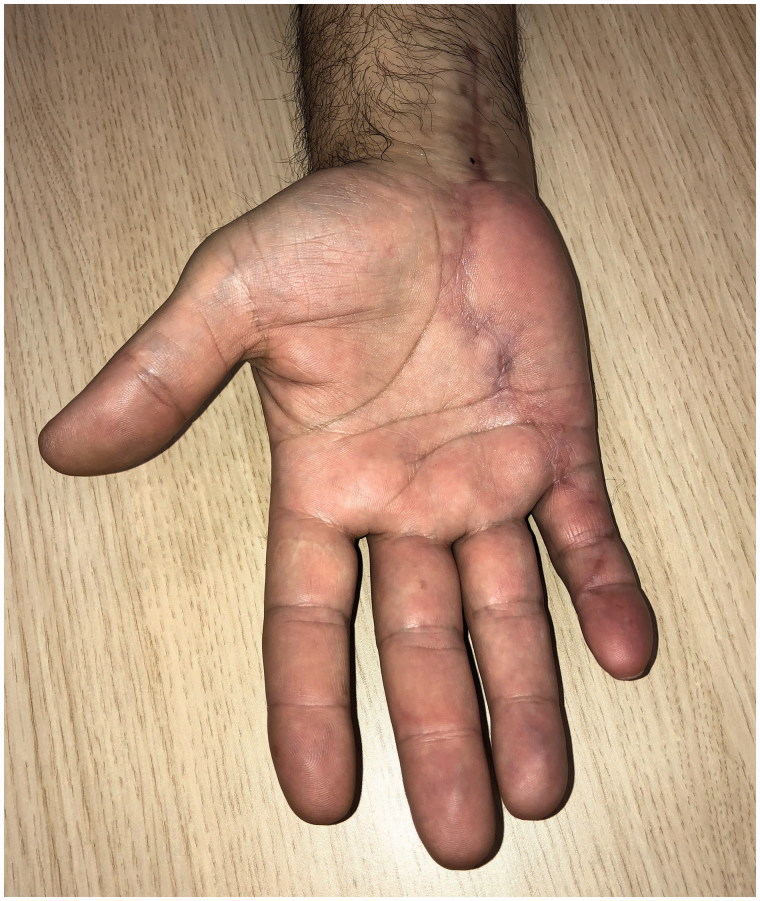
Full range of movement at three months.

**Figure 4.**
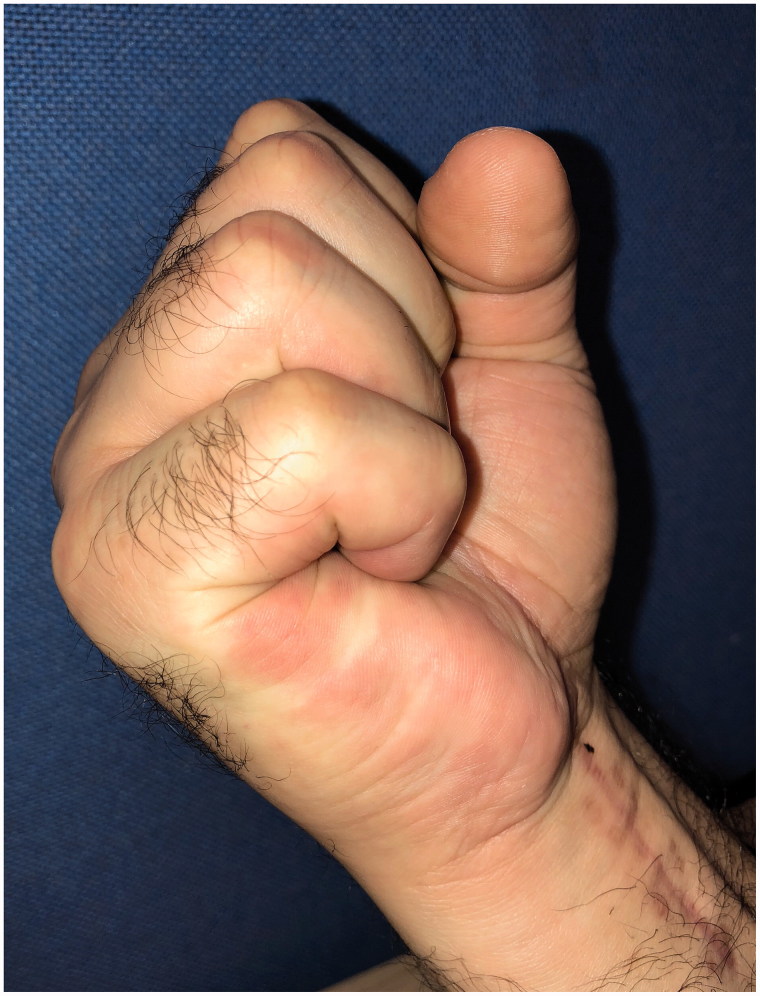
Full range of movement at three months.

Postoperatively, the patient underwent early active motion flexor tendon protocol with our hand therapy department. At three and six month follow up review, the tendon remained intact, he had regained his full range of motion and reported normal, pain free, grip strength (Figures 3 and 4).

## Discussion

FDP tendon avulsion at the bony insertion of the distal phalanx sustained during active flexion against resistance is common. This can be explained by McMaster’s work stipulating that the tendon is the strongest part of the musculotendinous unit [3]. Intra-tendinous rupture of FDP is rare and spontaneous rupture rarer with its mechanism poorly understood [1].

Boyes et al. described spontaneous rupture as intramembranous rupture without extrinsic or intrinsic pathology [1]. A case series by Bois et al. from 2007 presents five ‘spontaneous’ FDP ruptures [5]. As in our case, four of the five cases report rupture occurring both in the dominant hand and in the little finger with Naam et al. reporting that 92% of ruptures occur within the palm [5,6].

However, the cases presented by Bois et al. are Zone III ruptures unlike in our case where we present a Zone IV rupture [5]. An accompanying literature review by Bois et al. identified 50 spontaneous flexor tendon ruptures (i.e. FDS & FDP) in 43 cases over a 50-year period, of which only three of these were in zone IV illustrating the rarity of the case we present [5].

Boyes et al. presented 80 closed tendon injuries across a 13-year period with 68% of these injuries found at the musculotendinous junction and 32% occurring within the substance of the tendon [1,7]. Mechanisms described include blunt trauma, hyperextension and active flexion against resistance the mechanism described in our case [7]. The clinical presentation is diverse with a majority reporting a popping sensation, whilst others experience pain or cramping as in our case report [5].

The underlying pathophysiology in spontaneous rupture is unclear - it is likely to be multi-factorial involving changes at a microscopic level with studies by Kannus and Josza identifying mucoid degeneration, hypoxic degenerative and calcifying tendinopathy within all ruptured tendons when compared to healthy age-matched controls [2,7]. At the time of operative repair samples were not sent for histology and therefore we cannot comment on these microscopic changes. In future cases histological samples should always be sent.

In the case we present - avulsion was assumed at the insertion of FDP as this is a common injury. Intra-operatively, this was not found and the patient may have benefited from magnetic resonance imaging or ultrasonography pre-operatively to determine the level of rupture avoiding an extensive Brunner incision. Given the rarity of spontaneous FDP rupture within tendon substance in the palm (less than 1 case per year), the pickup rate for such rupture would be extremely low. Furthermore, the accuracy of US scan relies on the time from the actual injury [8].
